# Construction Materials Used in the Historical Roman Era Bath in Myra

**DOI:** 10.1155/2014/536105

**Published:** 2014-06-26

**Authors:** Cem Oguz, Fikret Turker, Niyazi Ugur Kockal

**Affiliations:** ^1^Vocational School of Technical Sciences, Akdeniz University, 07058 Antalya, Turkey; ^2^Department of Civil Engineering, Akdeniz University, 07058 Antalya, Turkey

## Abstract

The physical, chemical, and mechanical properties of mortars and bricks used in the historical building that was erected at Myra within the boundaries of Antalya Province during the Roman time were investigated. The sample picked points were marked on the air photographs and plans of the buildings and samples were photographed. Then petrographic evaluation was made by stereo microscope on the polished surfaces of construction materials (mortar, brick) taken from such historical buildings in laboratory condition. Also, microstructural analyses (SEM/EDX, XRD), physical analyses (unit volume, water absorption by mass, water absorption by volume, specific mass, compacity, and porosity), chemical analyses (acid loss and sieve analysis, salt analyses, pH, protein, fat, pozzolanic activity, and conductivity analyses), and mechanical experiments (compressive strength, point loading test, and tensile strength at bending) were applied and the obtained results were evaluated. It was observed that good adherence was provided between the binder and the aggregate in mortars. It was also detected that bricks have preserved their originality against environmental, atmospheric, and physicochemical effects and their mechanical properties showed that they were produced by appropriate techniques.

## 1. Introduction

The values that human beings have created either directly or with the nature since the beginning of human history until today within thousand years of history of civilization are today called “cultural and natural heritage.” Such values have been deteriorated and damaged because of the properties of soil where buildings are situated, use of defected construction materials, such internal reasons as mistakes in the construction design, and/or external reasons as fire, earthquake, and war. Preservation, repair, and reinforcing of historical buildings are important in terms of protecting the cultural heritage [1].

The original construction materials must be preserved in situ by the interventions to be made on historical buildings. When it is necessary to use new material, the materials, which are compatible with the original construction material in terms of physical, chemical, mechanical, and aesthetic properties, must be chosen. In order to choose new materials that are compatible with such properties, first the properties of the original construction materials must be defined [2, 3].

Mortars containing brick or tile powder and lime are called Khorasan mortar in the Ottoman and Cocciopesto in the ancient Roman time. Some researchers investigated the effects of pozzolanic materials such as ground granulated blast furnace slag and fly ash on Khorasan mortar composed of brick or tile powder and lime [4, 5]. Some researchers studied the characterization of Khorasan mortars applied to structures of certain Early Byzantine period and Ottoman time [6, 7]. From the results of the various analyses of the coating mortars performed by Labiadh et al. [8], it was determined that these consisted primarily of strongly carbonated air lime to which pozzolanic aggregates had been added. The purpose of another study was to trace a constructive history by means of chemical and mineralogical analysis of the mortars used [9]. The different binders and aggregates used, coming from different quarries, could thus suggest the existence of various moments of construction, with an expansion of the built structures.

Brick masonry constitutes a significant part of the construction materials found in historic buildings. Elert et al. [10] revealed that the results of their investigations could be used to evaluate the suitability of the bricks as replacements for original masonry materials and to predict their performance once in place. López-Arce et al. [11] determined chemical and mineralogical composition, texture, structure, and physical properties of bricks belonging to ancient buildings of Toledo City. The study was focused on selecting appropriate brick characterisation routines to be applied in the restoration of historical buildings. A previous study presented a review of the practice of analysing clay building bricks from Australian archaeological sites and its aim was to look at current archaeological practice and make suggestions about ways of improving the methods used with the intention of producing results that contribute to the understanding of the past [12]. Old clay bricks are difficult to characterize, due to the wide diversity of raw materials, manufacturing processes, and conservation states [13]. The physical, mechanical, and chemical properties of historical clay brick exhibit a large spectrum and a significant variability.

One of the important buildings, which is the most visible on surface belonging to Roman time at Myra, is bath. According to remainders of brick and mortar, Borchhardt dates 3rd century A.D. and Farrington dates 2nd or 3rd century A.D. Although some of the sections of the well preserved bath surrounded by the greenhouses are not appeared, five rooms can be identified today. It has classical Lycia bath plan. It is evident from the traces of walls that the bath was being heated from bottom and from walls. Wall is mostly made of brick mortar. Myra bath is specific for its construction material rather than its plan. It is the only bath which was fully built with brick in pure Roman technique [14].

The purpose of this study is to analyze the construction materials which were used in historical buildings in order to define their characteristic properties and to consider the properties of original materials, thus enlightening the usability of them in repair of such buildings. For this purpose, samples of the construction materials like mortar and brick, which were used at Myra bath within the boundaries of Antalya Province that was built during the Roman time, were taken and the characteristic properties of these materials were investigated.

## 2. Materials and Methods 

### 2.1. Materials Used in the Experimental Study

**Figure 1:**
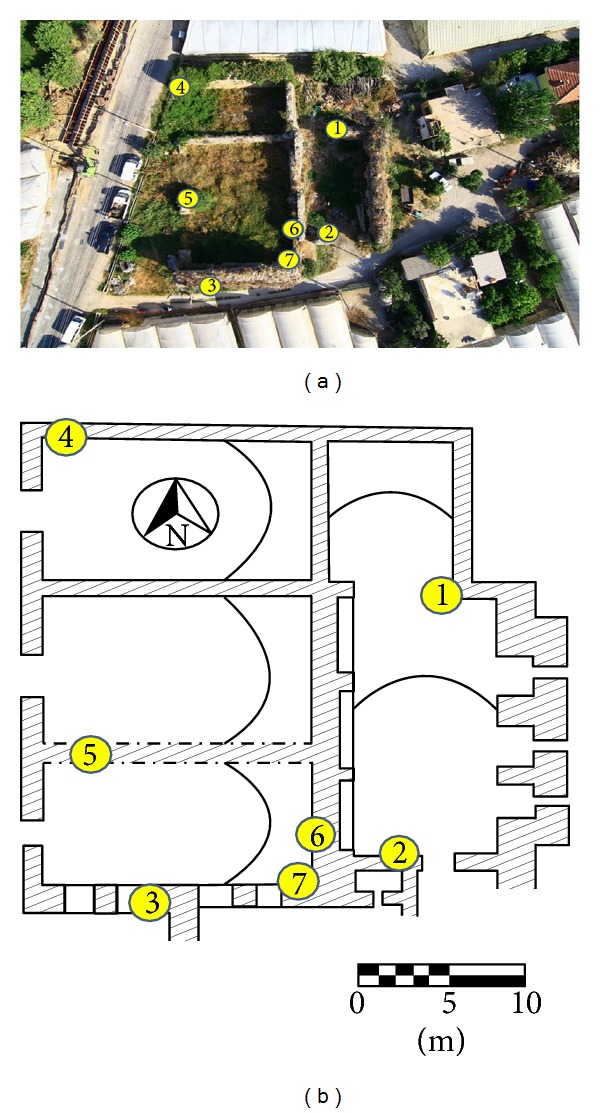
Locations where samples were taken on the 2011 aerial photo and plan of the bath (Myra-Andriake excavation archives).

5 brick and 6 mortar samples were taken from 7 different sections of the bath, located within the boundaries of Demre town of Antalya, within Myra antique city. The sections where samples were taken were marked on the plans and aerial photograph of the buildings (Figure 1) and such sections were photographed.

Attention was paid to the fact that samples were not taken from the surface as much as possible while taking wall mortar samples and it was the main principle not to damage original material. Therefore, instead of core drilling method, sampling with hand and other tools was preferred. Sampling operations were conducted in a great care under the supervision of the archeologists in the excavation team using hammer and chisel, not harming the building. Samples were taken out in mass as far as possible but it was not possible for some samples because they were weak and easily crumbled.

**Figure 2:**
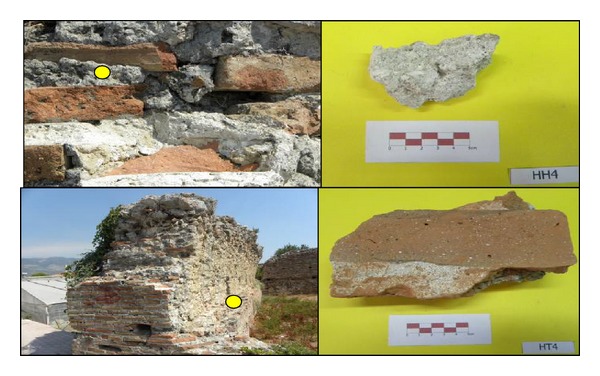
Brick wall mortar with code HH4 and brick wall sample with code HT4.

By means of macroobservations, shapes, dimensions, hardness, components, color, texture, density, and layers of materials were analyzed and the places where samples were taken and the samples were documented at macro- and microscale by photographing (Figure 2).

### 2.2. Macroscale Analysis

**Figure 3:**
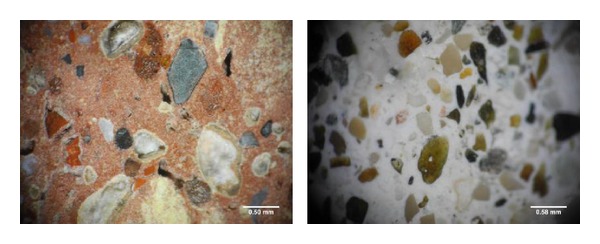
Evaluation of brick and mortar samples at macroscale.

The polished surfaces of samples were first visually inspected and then they were analyzed with the aid of trinocular stereo microscope with Nikon SMZ 745 T brand imaging apparatus and the scaled views of the samples are given in Figure 3.

Mortar had a hollow structure, containing rounded, semirounded, and/or cornered but mostly long and flat grains, from silt size approaching to diameter of 1 cm, especially in such colors of white, cream, black, dark, and reddish brown. We understand that most of the grains observed in mortar in the color of dark and reddish brown were crushed brick. However, we observed that large cracks were filled with the recrystallization of calcite minerals. We found that most of the various types of aggregates used in mortar were those having quartz, calcite, and feldspar mineral.

### 2.3. Physical Analyses

Physical analyses revealed important information about the characteristics of materials and conservation works. On the samples of brick and mortar taken from the bath, such physical experiments as water absorption rate (mass and volume) (*S*_*k*_, *S*_*h*_), unit volume mass (apparent density) (Δ), specific mass (real density) (*δ*), porosity (*p*), and compactness (*k*) were conducted according to the methods in TS EN 1936 [15]. Results are given in Table 1.

When the physical properties of mortar samples are considered, unit volume mass was 1.53–1.75 g/cm^3^, specific mass 2.36–2.57 g/cm^3^, water absorption rate in mass % 13.92–21.73, water absorption rate in volume % 24.37–33.52, porosity % 30–41, and compactness % 59–70. These results showed that mortars were hollowed and therefore light.

When the physical properties of brick samples are considered, unit volume mass was 1.80–1.86 g/cm^3^, specific mass 2.50–2.79 g/cm^3^, water absorption rate in mass % 13.26–16.68, water absorption rate in volume % 24.59–30.59, porosity % 26–34, and compactness % 66–74.

The literature survey and the authors' tests of bricks indicate a high porosity (15–40 vol.%) and water absorption (10–20 vol.%). The suction can be rather high (up to 0.35 g/cm^2^/min), while the apparent density is low (1.5–1.8 g/cm^3^). The compressive strength shows a huge scattering with values mostly ranging from 1.5 to 30 MPa. No trends could be found regarding age or origin, as the amount of data is limited [13].

### 2.4. Chemical Analyses

#### 2.4.1. Acid Loss and Sieve Analysis

Acid loss analysis was conducted in order to separate binding fine and coarse aggregates in mortar and to calculate binding lime amount. Though this analysis gives no healthy results because it causes dissolution in limestone origin aggregates, separation was made using a mallet to ensure exposition of the binding part to acid as far as possible. The part that did not react with acid and was retained on the filter paper was dried in the oven and loss in acid was determined. The results are given in Table 2. It was observed that binder-aggregate ratio in mortar varied between 1/6 and 1/8.

**Figure 4:**
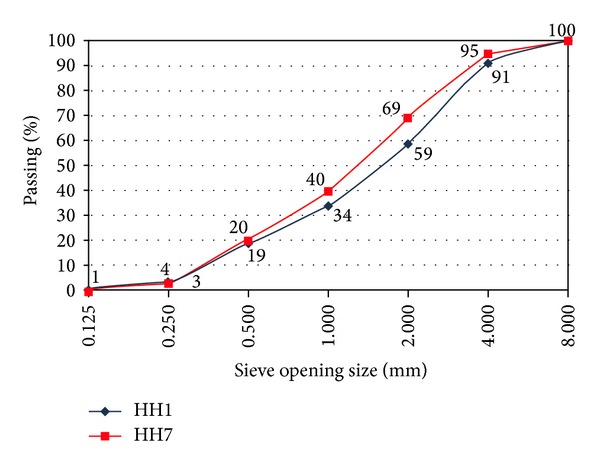
Sieve analysis of the aggregates in the mortar samples with codes HH1 and HH7.

Results of the sieve analysis that was conducted in order to determine aggregate particle size and distribution in historical mortar samples are given in Figure 4. It was found that maximum particle size of aggregate was 8 mm. Coarse aggregates in mortar were rounded whereas fine aggregates were angular/cracked. This shows that the aggregates taken from the sea and the rivers were used together in the mortars. On the other hand, Bartz and Filar [16] indicated that observed differences in the composition of the filler, as well as grain-size distribution, were not accidental, but resulting from different function of mortars.

#### 2.4.2. Salt Analysis

Salt analyses covering determination of chloride, nitrate, sulfate, and carbonate ions were conducted in order to find out existence of salt in the samples, which caused deterioration in the materials. These analyses were measured relatively and shown as + − in the table. If amount of salt found during analysis was high, its + value was also increased. Salt analysis results of the mortar samples are shown in Table 3. Chlorine was detected in the HH1 sample from the external region of Myra bath. It is thought that chlorine comes from seawater. It is held that sulfate and carbonate would exist in HH1 sample due to agricultural waste occurring from active agriculture around the bath and the atmospheric pollution because it was exposed to in-city traffic. We hold the opinion that nitrate in the samples was originated from agricultural waste because the region had intense agricultural activity.

#### 2.4.3. Conductivity, pH, Protein, and Fat Analyses

There are various methods available for determining the pozzolanic activity in the literature and these are classified as direct and indirect methods [17, 18]. Pozzolanic activity in mortar samples was found out by measuring electrical conductivity [2, 6]. If difference between conductivities is bigger than 1.2 mS/cm, mortar is good pozzolana, if the difference is between 0.4 and 1.2 mS/cm, mortar is pozzolana, and if it is smaller than 0.4 mS/cm, mortar is not pozzolana [7, 19]. As seen in Table 4, because the difference between conductivities was found to be 0.40–0.85 mS/cm in 5 of the 6 samples analyzed (83%), they had pozzolanic activity. In the remaining 1 sample (17%) the difference between conductivities was smaller than 0.40 mS/cm and therefore it was not pozzolanic.

pH value was measured in order to find out if the materials were acidic or alkaline. It was expected that mortars would have weak alkaline properties because lime was strongly alkaline and the value between 8.23 and 8.98 as seen in Table 4 confirmed this. We understand that samples completed the process of silication and carbonation.

Protein and fat analyses were conducted in order to determine organic additives in the samples taken from historical buildings. Though it is thought that protein in the samples generally originates from protein based additives like egg, blood, casein, cotton waste, plant fiber, and animal hairs, it may be due to polluted materials. As seen in Table 4, out of the 6 samples analyzed, protein was detected in 4 samples (67%), fat was detected in 5 samples (83%), and both protein and fat were detected in 4 samples (67%). It is thought that they were additive-originated used in mortars and/or polluted materials. However, when the possibility of deterioration of protein in time is considered, it is difficult to make a decision for the samples of which protein existence could not be detected.

As seen in Table 5, because the difference between conductivity is 0.40-0.41 mS/cm in two of the five brick samples analyzed, they had pozzolanic activity. The other three samples were not pozzolanic because the difference between conductivity was less than 0.40 mS/cm. These values show that bricks had pozzolanic substance in low quantity. This result indicates that the amount of clay minerals used in the production of bricks was not sufficient to produce pozzolanic amorphous material in high quantities. pH values of bricks are between 11.33 and 11.44 as seen from Table 5.

### 2.5. Microstructural Analyses

In addition to the determination of physical, chemical, and mechanical properties of bricks and mortars, determination of their microstructural properties is also of importance. Microstructural analyses will be required in the investigation of the compliance of the materials to be used for repair with original raw materials.

#### 2.5.1. SEM/EDX Analysis

**Figure 5:**
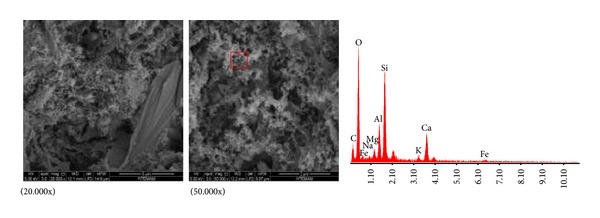
HT4 brick SEM/EDX images with magnitude.

SEM/EDX analyses of HT4 brick sample revealed silica, aluminum, calcium, and iron in high percentages and magnesium, potassium, and sodium in low percentages (Figure 5, Table 6). It was determined by the chemical composition analyses conducted with EDX that the bath (3rd century) contained silica, aluminum, calcium, and iron in high percentages and magnesium, potassium, and sodium in low percentages. Bricks contained high percentage of iron. It can be explained that bricks were produced by using more clay, which contained high percentage of iron oxide. If use of such clay was deliberately made, the reason may be that high iron content has positive effects on sintering properties. Though the bricks used in the bath and the theatre at Myra were produced in different centuries, their similar ingredients showed that the raw materials were taken from the same source if less change had been made in their compositions. It is possible to say that the temperature of bricks to be vitreous is between 800 and 1000°C. We observed from scanning of brick samples with electron microscope (SEM) that no vitreous structure was formed. This verifies that bricks were fired at low temperatures. Invisibility of the mullite peaks at XRD analyses, which were formed at high temperatures, indicated that temperature did not exceed 900°C [20]. Bricks were not in homogenous structure and contained coarse natural aggregates and brick crumps.

The mortar of Roman age that was used at Myra bath was composed of calcite and quartz minerals. Calcite mineral shows that lime was used as binding substance. On the other hand, quartz mineral shows that the aggregates used generally contained silicon mineral. High percentage of calcium as found in the analyses conducted with SEM/EDX shows that pure lime was used in the preparation of mortar. In the SEM images of mortars, it was observed that the aggregates and the mortar were properly bonded with structures made of needle-like texture in their interface. In the chemical composition analyses of interfaces made with EDX, calcium, silicon, and aluminum elements were observed. Amount of calcium in mortar matrix and amount of silicon in aggregates were more than interfaces. These results show that lime and pozzolanic aggregates reacted and formed hydraulic products, namely, calcium silicate hydrate (C-S-H) and calcium aluminate hydrates (C-A-H) at interfaces. Formation of such products gave high mechanical properties to mortars. The results obtained from these analyses showed parallelism with other results of previous investigation [21].

The fact that the aggregates used in mortars contained high percentage of silicon dioxide and aluminum oxide, which show pozzolanic property, as found in EDX analyses (HH4) showed that calcium silicate hydrate and aluminate hydrate structures were formed in the interface of lime and aggregates, which promoted strength. The extended form among calcite crystals seen in the SEM images taken from binding section of mortars may be interpreted as formation of calcium silicate. Limestone, which is the raw material of lime, is sedimentary rock containing high percentage of calcite minerals. Limestone usually contains dolomite. Dolomite is a sort of mineral containing calcium and magnesium carbonate (CaMg(CO_3_)_2_). If percentage of dolomite in limestone is higher than 50%, it is called limestone, if percentage of dolomite in limestone is between 10 and 50%, it is called dolomitic limestone, and if it is lower than 10%, it is called natural limestone [22]. The fact that percentage of magnesium did not exceed 10% in the binding section of mortars at EDX analyses and the (CaMg(CO_3_)_2_) phase was not properly visible at XRD analyses because of low quantity showed that the lime used as binding agent in mortars was obtained from natural limestone. Also, existence of carbon elements in SEM/EDX analyses indicated that organic additives were used (herbal and animal origin) in the mortar in order to promote rheology and mechanical properties of the samples [23]. As seen in Table 4, existence of protein and fat in 5 samples of the 6, which were tested, supports this fact.

#### 2.5.2. XRD Analysis

**Figure 6:**
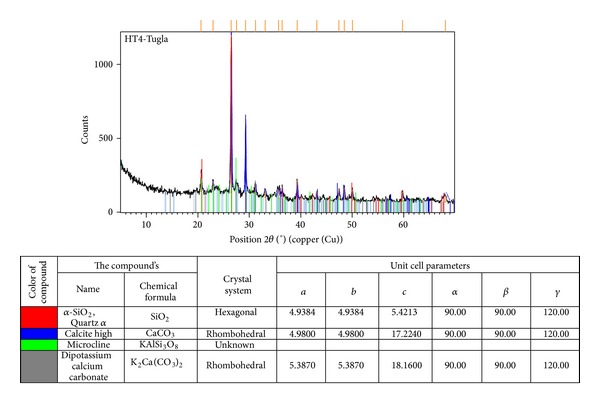
HT4 brick sample XRD graph and description table.

Further to the XRD analyses, conducted on total 11 mortar samples, similar samples were specified and name of components, chemical formula, crystal systems, and unit cell parameters of 3 samples were identified (Figure 6).

In all brick samples, quartz, various feldspars, and calcite minerals were observed. The fact that calcite (CaCO_3_) peaks were detected in almost all samples assisted in estimating that bricks were fired at temperatures between 750 and 900°C [24]. The minerals detected by XRD give information about the firing temperatures of bricks. Firing of bricks at high temperatures (≥900°C) caused deterioration at their amorphous structures and formation of such high temperature products like spinel, mullite, and cristobalite. The fact that no such minerals as mullite and cristobalite were detected in the X-ray diffraction patterns of 5 bricks and illite mineral was observed shows that the firing temperatures of these bricks did not exceed 900°C. Existence of hematite indicated that the bricks were fired around 850°C. Because the anorthite mineral, found in HT2 brick sample, was detected in high temperatures, we can say that the bricks were fired in higher temperatures when compared with others. The illite minerals detected in XRD analyses indicated that these bricks had weak pozzolanic properties. It was indicated that illite minerals usually have negative effect on pozzolanic property [25]. As seen in Table 5, the fact that 2 of the 5 brick samples analyzed had pozzolanic activity because the difference in conductivity was 0.40-0.41 mS/cm and the remaining 3 samples had no pozzolanic activity because the difference in conductivity was smaller than 0.40 mS/cm verified this fact. The increase at slope observed in 20–30 degrees, at 2*θ* interval of diffraction pattern of bricks, indicated existence of amorphous structures.

### 2.6. Mechanical Analysis

Because mortar samples were not in standard sizes, compressive strength test could not be performed and therefore point load test was preferred. Point load tests were conducted on 3 mortar samples. Results are shown in Table 7. The ratio between the point loading strength index and uniaxial compressive strength is called strength conversion factor index (*K*) [6, 26, 27]. For *K* value, the value 10,6471 that is valid for weak rocks was taken [28]. Equivalent uniaxial compressive strength value was obtained by multiplying this value by corrected point load index.

The compressive strengths based on the results of point load test were defined as 17.4 MPa in average at the bath (3rd century). The reason why compressive strength was found high may be that such parameters as steam and heat were existent in a place like bath. Flexural strength and compressive strength tests were conducted on 5 brick samples. Results are given in Table 8.

According to the results of flexural strength test of brick samples, 4.9 MPa was found in average in the bath (3rd century). According to the results of compressive strength test of brick samples, 33.5 MPa was found in average in the bath (3rd century). The compressive strength values of Roman period bricks (HT2, HT3, HT4, HT5, and HT6) fulfill the minimum compressive strength value under TS EN 771-1 [29] given for medium strength clay brick.

## 3. Conclusions

The mortar and bricks used in the historical buildings are historical documents because they give important information about the construction technology of the period in which they were used. Analysis of historical construction materials will reveal useful information about the techniques of how such construction materials are prepared, the civilizations where they are used, and the progress and the phases that such civilizations have undergone until today.

Following conclusions can be drawn from the experimental studies and analyses mentioned above.

The mortars used at Myra bath were composed of calcite and quartz minerals. Existence of calcite mineral showed that lime was used as binder. On the other hand, existence of quartz mineral showed that the aggregates mainly contained silicon mineral. It was detected that mortars had low unit weight and high porosity.

It was observed from SEM analysis that good adherence was provided between the binder and the aggregate. Existence of high percentage of calcium in SEM/EDX analyses of mortar showed that pure lime was used in the preparation of mortar.

As observed in SEM images of brick samples, no vitreous structure was formed. This verified that bricks were fired at low temperatures. Absence of such minerals as mullite and cristobalite in the X-ray diffraction patterns of bricks and observation of illite mineral indicated that firing temperatures of bricks did not exceed 900°C.

As a result, the information obtained from this study will enlighten the works of protection, repair, and strengthening of historical buildings in the future. They will lead the investigators who will carry out researches on historical buildings in countries which have a rich cultural heritage.

## Figures and Tables

**Table 1:** Results of physical analyses of brick and mortar samples.

SN	Sample code	Δ (g/cm^3^)	S_k_ (%)	S_h_ (%)	δ (g/cm^3^)	Porosity (%)	Compactness k (%)
1	HT2	1.82	13.60	24.79	2.60	30	70
2	HT3	1.86	14.43	26.90	2.63	29	71
3	HT4	1.80	15.46	27.80	2.68	33	67
4	HT5	1.83	16.68	30.59	2.79	34	66
5	HT6	1.85	13.26	24.59	2.50	26	74
6	HH1	1.75	13.92	24.37	2.50	30	70
7	HH2	1.63	18.21	29.63	2.50	35	65
8	HH3	1.59	20.29	32.25	2.36	33	67
9	HH4	1.56	21.29	33.22	2.38	34	66
10	HH5	1.53	21.73	33.16	2.57	41	59
11	HH7	1.61	20.81	33.52	2.48	35	65

**Table 2:** Acid loss analysis results for historical mortar samples.

SN	Sample code	Percentage of loss in acid (%)	Binder/aggregate ratio
1	HH1	11,7	1/8
2	HH7	14,3	1/6

**Table 3:** Salt analyses in mortar samples.

Sample code	Sample type	Chlorine Cl^−^	Sulfate SO_4_^−2^	Carbonate CO_3_^−2^	Nitrate NO_3_^−^
HH1	Mortar (brick_brick)	++	−	+	+
HH2	Mortar (brick_brick)	−	−	−	−
HH3	Mortar (brick_brick)	−	−	−	+
HH4	Mortar (brick_brick)	−	−	−	−
HH5	Mortar (brick_brick)	−	−	−	+
HH7	Mortar (brick_brick)	−	−	−	−

**Table 4:** Conductivity, pH, protein, and fat analyses in mortar samples.

Sample code	Sample type	Conductivity (mS/cm)	pH	Protein	Fat
HH1	Mortar (brick_brick)	0.41	8.23	−	+
HH2	Mortar (brick_brick)	0.79	8.74	+	+
HH3	Mortar (brick_brick)	0.63	8.80	+	+
HH4	Mortar (brick_brick)	0.65	8.98	+	+
HH5	Mortar (brick_brick)	0.34	8.78	−	−
HH7	Mortar (brick_brick)	0.48	8.94	+	+

**Table 5:** Conductivity and pH analyses of brick samples.

Sample code	Sample type	Conductivity (mS/cm)	pH
HT2	Brick	0.21	11.34
HT3	Brick	0.09	11.35
HT4	Brick	0.41	11.44
HT5	Brick	0.40	11.41
HT6	Brick	0.29	11.33

**(a):** 

Element	C	O	Na	Mg	Al	Si	K	Ca	Fe
Wt %	12.92	37.97	1.14	2.26	7.29	18.28	1.76	14.15	4.25

**(b):** 

Oxide	Na_2_O	MgO	Al_2_O_3_	SiO_2_	K_2_O	CaO	Fe_2_O_3_
Wt %	1.81	4.37	16.02	45.58	2.44	22.8	6.98

**Table 7:** Results of mortar sample point load test.

SN	Sample code (century)	Sample type	*I*_*s*_ = *P*/De^2^	*I*_*s*_(50) = *F* · *I*_*s*_	Compressive strength (MPa)
1	HH2 (3)	Mortar	5.74	2.13	22.7
2	HH3 (3)	Mortar	3.07	1.21	12.9
3	HH5 (3)	Mortar	4.37	1.57	16.7

**Table 8:** Results of flexural strength and compressive strength of brick samples.

SN	Sample code	Flexural strength (MPa)	Compressive strength (MPa)
1	HT2	5.2	47.8
2	HT3	8.2	23.3
3	HT4	4.7	44.0
4	HT5	3.8	32.3
5	HT6	2.8	20.0
